# The Lived Experience of Play and How It Relates to Psychological Wellbeing: An Interpretive Phenomenological Analysis (IPA) Study Amongst Undergraduate Students from Medicine, Nursing, and Allied Health Professions' Programmes in the United Kingdom

**DOI:** 10.1155/2024/7871499

**Published:** 2024-04-03

**Authors:** Rebecca Rylance-Graham

**Affiliations:** University of Liverpool, School of Health Sciences, The Quadrangle, Brownlow Hill, Liverpool L69 3GB, Merseyside, UK

## Abstract

Current literature acknowledges that undergraduate students undertaking programmes in medicine, nursing, and allied health professions experience occupational stress which presents as a detriment to mental health, psychological wellbeing (PWB), and burnout. Strategies to improve the wellbeing of students have been slow to embed and have had limited impact, indeed the issue of declining wellbeing amongst this group is escalating. Studies from the business literature suggest that organisations that foster a playful environment reap benefits in terms of employee wellbeing. This interpretive phenomenological analysis (IPA) study explored the lived experiences of play amongst undergraduate students from medicine, nursing, and allied health professions' programmes in the clinical practice setting. The resultant findings offer some unique empirical insights into the types of play that the students engaged in, ranging from informal banter with peers and patients to artful, sophisticated, cocreated play. The study also revealed insights about the factors which facilitate play, notably the “big personalities” on the ward. The factors which limited play are related to the tension between being a health professional and the enactment of play as well as hierarchical factors. Crucially, the study found that the practice of play induced key hedonic and eudaimonic PWB benefits to the students, ranging from positive affect to improved relationships, a sense of meaning, and a positive learning environment, offering original empirical insights. These findings have not been observed previously and shine a conceptual light on a previously unknown phenomenon.

## 1. Introduction

For many decades, the mental health and wellbeing of UK healthcare workers have attracted attention as a major public health concern. Indeed, employee wellbeing has been the focus of many organisational imperatives [1–3]. Despite the somewhat nebulous term, the concept of wellbeing stems from the positive psychology movement [4]. Considered to be a dynamic and multifaceted continuum [5], PWB is frequently conceptualised within psychology literature as a combination of positive affective states [6] and is rooted in the hedonistic and eudaimonic traditions. The hedonic tradition relates primarily to affective states such as achieving a balance between positive and negative emotions and feeling happy and satisfied in life, whereas the eudaimonic perspective is concerned with the fulfilment of human potential and a meaningful life [7]. To put simply, wellbeing relates to feeling good and flourishing in life [8, 9].

Since the SARS-CoV-2 virus (COVID-19) pandemic, the wellbeing of those who provide front-line care is at a crisis point [10, 11]. Of further concern is an often-disregarded workforce population, healthcare students. Students undertaking clinical programmes (degree programmes which lead to professional registration with a regulatory body) are amongst the most vulnerable occupational groups with regard to poor wellbeing and mental health [12]. This is largely due to the fact that they are located within two distinct organisational contexts: higher education and clinical practice. A combination of academic workload and responsibilities in clinical practice is thought to contribute to a significant psychological burden amongst this group [13]. With mounting referrals to university counselling and occupational health services, the wellbeing of the UK student population is at a crisis point [14]. Crucially, since the COVID-19 pandemic, the mental health and wellbeing of undergraduate students have reached a critical juncture, with a recent report by the Office for National Statistics (ONS) [15] stating that students reported unprecedented levels of anxiety, unhappiness, and life dissatisfaction.

Strategies to improve PWB before and during the pandemic tended to focus on prevention and self-management, with targeted support such as education, counselling, resilience training, and meditation/mindfulness [16, 17] (NHS England and NHS Improvement, 2021). However, there is limited evidence that such strategies produce favourable outcomes [18], suggesting perhaps that there may be other previously unexplored strategies to enhance PWB.

The subject of play, for example, amongst the workforce is an emerging topic of research inquiry. Studies have shown that play can affect the emotional climate in the modern workplace [19] and that organisations which foster a playful work environment and employees who engage in the playful practice benefit greatly in terms of enhanced PWB [20]. Studies suggest that the practice of play appears to generate hedonic and eudaimonic wellbeing benefits to both employers and employees, bringing about increased engagement, enhanced productivity, improved job satisfaction, and a reduction in workplace stress and burnout [21–26].

That withstanding, extant play studies have been almost entirely conducted within the business sector and there are limited studies which have explored the playful practice of those who work within the healthcare organisational setting. Furthermore, there is a dearth of studies which have explored the practice of play amongst undergraduate students undertaking clinical programmes. This study seeks to address the gaps in the literature by exploring how play is expressed amongst undergraduate medicine, nursing, and allied health professions' (AHP) students and how it influences psychological wellbeing.

## 2. Literature

There are many definitions of play, and the distinct and sometimes discreet characteristics of play, for example, joking, humour, and playing games, make it difficult to achieve a universally accepted definition [21]. Indeed, there is much debate within the literature about the definition of play in the context of the workplace, and a consensus concerning the definition of organisational play is yet to be established. However, Celestine and Yeo [27] succinctly define play in the organisational context as follows:

“Activity undertaken in a work context that is interactive in nature and undertaken with the goal of having fun” [27].

Characterised by fun, humour, competition, and fantasy, play is often regarded as the activities which exist outside the confines of the workplace [28]. Indeed, until recently, work and play were considered to be two distinct and incompatible domains [29].

Perhaps due to the ostensibly nonserious and light-hearted image of the play, the concept of play at work and the potential benefits have remained a relatively understudied topic of research inquiry [21]. Contemporary thinking challenges the work/play dichotomy as something of a misnomer and asserts that it is possible to integrate the two in order to benefit individuals and organisations [21, 28, 30].

Current literature acknowledges that students undertaking programmes in medicine, nursing, and AHPs experience occupational stress which presents as a detriment to mental health, wellbeing, and burnout [12]. Stress in student nurses, for example, has been associated with depression, anxiety, and impaired emotional wellbeing [31, 32], and whilst much of the extant literature is located in the nursing press, there are studies which have shown that medical students also experience stress when faced with exam pressures or when caring for sick patients for the first time [33]. Similarly, students undertaking AHP programmes such as physiotherapy and radiography also experience occupational stress, which presents as poor PWB and burnout [34, 35]. Burnout levels amongst this group of students are high and have been linked to increased suicidal ideation, reduced self-esteem, and programme attrition [36].

That aside, studies have shown that healthcare workers (both students and staff) do engage in a number of practices which help them cope with stress and improve psychological wellbeing (PWB). Activities that provide a sense of joy, having fun, and utilising social support systems have been cited in the literature [37, 38]. These “informal” playful practices are not well understood, yet capture the playful activities which would seem to address PWB. Take, for example, the practice of humour and jocularity, having a sense of humour is thought to be a vital job resource for healthcare professionals and a crucial buffer against organisational adversity [39]. Indeed, there is a long tradition of a “gallows humour” amongst clinicians [40]. Gallows humour relates to the practice of mocking in times of extreme adversity such as death and dying [41]. Argued by some as being inappropriate, unprofessional, and disrespectful, and by others as a paradoxical, yet is a necessary human function to cope with the enormity of illness, disease, and death [42]. Studies have shown that healthcare workers who engage in humour in the workplace are more likely to achieve job satisfaction and are less likely to leave their profession [43, 44]. Furthermore, the link between humour and positive wellbeing is well-established within the existing literature [39, 45]. Humour and other expressions of play appear to provide psychological benefits for healthcare workers during times of stress or crisis [46].

Notwithstanding, the organisational play literature alludes to an apparent synergistic relationship between play, the achievement of organisational imperatives, and PWB. However, there are no extant studies which have empirically explored play as a conduit for improved PWB, thus exposing a gap. There are also gaps in the literature in relation to the drivers of play, and studies have tended to focus on employer-driven play, with the informal playful practice of employees remaining largely unknown. Similarly, the factors which facilitate or limit play have gone unnoticed in the literature.

## 3. Research Question

“How is play expressed amongst undergraduate students from medicine, nursing, and allied health professions' programmes and how does it relate to psychological wellbeing?”

## 4. Research Aim

To provide an in-depth exploration of the lived experience of play in the clinical environment amongst medicine, nursing, and AHP undergraduate students.

## 5. Research Methods

### 5.1. Methodological Approach

Since the aim of the study was to explore the lived experiences of play in the clinical environment, the study employed a qualitative phenomenological design through the use of the structured approach embedded within the interpretative phenomenological analysis (IPA). IPA draws from three distinct philosophical stances: phenomenology, hermeneutics, and idiography [47]. IPA is a fitting approach to inquiry as it is congruent with the topic of exploration and it was anticipated that the depth of interpretation and faithfulness to the unique experiences of the participants could not have been gleaned by adopting other qualitative approaches.

### 5.2. Sample

As the topic of inquiry relied on participants who could talk in-depth about their unique experiences of play in the context of the healthcare environment, participants were recruited from a nonprobability sample from the Schools of Medicine and Health Sciences at a United Kingdom university. Only 3rd year undergraduate students, those who had experienced the most clinical practice, were recruited. There were nine participants in total: (male = 2; female = 7) Participants were recruited from Medicine (*n* = 1) Nursing (*n* = 4), Physiotherapy (*n* = 2), and Therapeutic Radiography programmes (*n* = 2). Each participant was allocated a pseudonym to protect their identity (see Table 1).

### 5.3. Data Collection

Data were gathered using in-depth 1 : 1 semistructured interviews in order to glean rich, meaningful descriptions of the participants' lived experiences of play within the clinical setting. Interviews were guided by an interview topic guide (see Table 2 overleaf).

Consistent with the interpretative phenomenological analysis (IPA) studies, the topic guide did not dictate the interview but instead served as a guide to facilitate flow and prompt narrative of the topic area. Each interview took place online or on campus (adhering to local and national social distancing rules at the time) and audio was recorded before being transcribed verbatim. Interviews took place over a period of five months and lasted between twenty-one minutes and fifty-eight minutes (mean: 40.7 minutes).

### 5.4. Data Analysis

Since the aim of the study was to explore the lived experiences of the enactment of play and how it relates to PWB, the study employed a qualitative phenomenological design, through the use of the structured approach embedded within interpretative phenomenological analysis (IPA). This facilitated the detailed exploration of the participants' lived experiences, allowing the researcher to explore and interpret the unique and nuanced perspectives of play in the clinical setting. The distinguishing elements of IPA, in contrast to other phenomenological approaches, lie in the meticulous process of analysis. Smith and Nizza [48] and Smith et al. [47] offer a procedural approach which informed the study design.

Each step of the analytical process necessitates full immersion in the data [49]. The purpose of the reading and rereading stage was to ensure that the participant remained the focus of the analysis [47]. Reading and rereading the transcripts facilitated exploratory noting (EN) and “free coding” [50]. Each participant's narrative account was distilled into a personal, unique experiential statement (ES). Each ES was subsequently tabulated, with a supporting participant narrative resulting in a case-level summary for each participant paying attention to the ideographic nature of IPA to ensure that the participant's “voice” had not been lost in the process. By searching for patterns and themes, the initial experiential statements (ESs) were then clustered into personal experiential themes (PETs). The process was then repeated for each participant. The resultant PETs were compared across cases to identify which PETs were most potent across all datasets. By searching for connections and clusters across cases, the data were reduced without losing the unique experiences of the participants. From an ontological perspective, it was imperative that the themes were rooted in the participants' lived experience of play. The subsequent PETs were clustered to provide a final master table of group experiential themes (GETs) (see Table 3 overleaf) and are thus presented as three GETs and are ordered to satisfy the research question as follows:

“How is play expressed amongst Undergraduate Students from Medicine, Nursing and Allied Health Professions programmes and how does it relate to Psychological Wellbeing?”

### 5.5. Quality

Unlike quantitative research which has a range of well-established methods and conventions for promoting trustworthiness, it must be acknowledged that the notion of trustworthiness in qualitative research inquiry remains a topic of much scholarly debate [51] and there is a lack of well-defined criteria within which to judge the quality of qualitative research. Arguably, the diligent step-by-step process of analysis required in the undertaking of an IPA study, highlighting the transparent and complex analysis will demonstrate quality [47]. Yardley [52], however, proposes that the three characteristics of (1) sensitivity to context, (2) commitment, rigour, transparency, and coherence, and (3) impact and importance demonstrate quality in qualitative studies.

#### 5.5.1. Sensitivity to Context

The context of the study embraces multiple facets such as awareness of the extant literature, theoretical underpinnings, and prior, established methodical approaches. Whilst knowledge of this may influence interpretation, it is essential that the analysis remains faithful to the data.

#### 5.5.2. Commitment, Rigour, Transparency, and Coherence

Commitment refers to the prolonged engagement with the topic of inquiry and the development of the researcher, as they advance their skills in the undertaking of the analysis. Rigour encompasses the completeness of the study in terms of data collection and analysis. Maintaining reflexivity by providing a clear audit trail sharing of notes, transcripts, and coding procedures with the supervisory team supported transparency.

#### 5.5.3. Impact and Importance

As a much-misunderstood topic of inquiry within the healthcare context, it is envisaged that the empirical findings from the study will offer unique insights into the expression of play as a conduit of improved PWB amongst medicine, nursing, and AHP undergraduate students. Moreover, it is hoped that the findings will add to a sparse body of literature and persuade further critical discussion among the academic and health community.

### 5.6. Ethics

Permission to undertake the research was granted by the University of Chester Business' School Ethics Committee (19/11/2021) and the University of Liverpool' Research Ethics Team (29/11/2021).

## 6. Findings

The resultant data revealed common themes which spanned a wide range of playful experiences encountered by the participants during the undertaking of their clinical placements. Their collective experiences revealed a number of factors related to play and PWB and the features which facilitated or limited play. In keeping with the IPA tradition, examples are shared from each participant, demonstrating commonality across cases yet maintaining the ideographic focus of each participant.

Through the detailed analysis of the participants' experiences, it was clear that they all engaged in playful practice during their clinical placements. Frequently expressed as fun, pranks, banter, and jocularity, each participant revealed aspects of play which aligned with Seligman's [8] PERMA wellbeing indicators. Seligman's PERMA flourishing model of wellbeing which encompasses positive emotion, engagement, relationships, meaning, and accomplishment reflects the essential components of flourishing. Furthermore, flourishing is associated with reduced stress, improved health, and the promotion of resilience [53].

## 7. GET (1): Playtime

Playtime was created to capture the participants' lived experiences of playful practice during their clinical placements. This GET illuminates the reader to some of the types of play that the participants engaged in. Since the practice of play among medicine, nursing, and AHP students is rarely discussed in the literature, it was necessary to create a shared and lived account of how play is expressed in the clinical environment. It was evident from the analysis that every participant had engaged in play during the undertaking of their clinical placements. Each participant shared their unique experiences of play; the subthemes reflect the nuanced and sometimes discreet elements of play.

### 7.1. Subtheme (1): Informal Play

The majority of the contemporary literature related to organisational play is dominated by serious play, which is ostensibly employer-driven. Furthermore, the findings from the literature review exposed informal play as a much-neglected area of academic inquiry. Since the expression of informal play in the healthcare organisational context remains unknown, the findings from the study revealed some powerful insights about the types of informal play that the participants engaged in. This subtheme is organised into two distinct sections: “unstructured” and “structured,” to capture the often spontaneous and nuanced aspects of informal play.

#### 7.1.1. Unstructured Informal Play (Banter, Teasing, and Pranks)

Unstructured informal play refers to the spontaneous employee-driven aspects of play which were free from structure or any play props such as games or prizes. All participants shared their experiences of having humorous banter with peers, qualified staff, and patients. According to the Oxford English Dictionary [54], “banter” is defined as “teasing, joking, or humorously mocking remarks exchanged playfully with another person or group.” Alternative definitions suggest that banter is a form of jocular abuse [55]. Popularised as common vernacular amongst young people, banter has established itself as a means of expressing humour and promoting social bonding [56]. Banter, as unstructured, informal play was experienced by all participants and was frequently expressed as teasing and pranking.

Penny shares her experience of theatre staff teasing her following an event where she had fainted in the theatre as follows:

“And then in the next surgery they were like “don't fall over in this one” kind of thing, so they were like poking fun at that, which was nice kind of affectionate” (Penny p11).

Penny associates the teasing practice of her peers as being psychologically affirming. Similarly, Leon described how teasing amongst staff about borrowing and returning equipment could be a funny experience. He also alluded to the notion of the staff being “down in the dumps.” Here, it would seem that Leon believed that this was a shared feeling amongst healthcare workers and that the enactment of play brought about psychological benefits.

“So, erm, I feel like when you go over and you like ask to borrow something they're like “make sure you bring it back” but sometimes you'll have a good joke about like just borrowing equipment, I don't know if that shows like how down in the dumps, we all are, but something just making jokes about little things like that can be so funny sometimes” (Leon, p12).

Both participants attributed the enactment of banter as being a positive playful event, initiating and confirming relationships and adding value to their clinical placement experience.

Related to the banter was the topic of food. Most participants described food as being a playful experience, and whilst the food in and of itself was not necessarily playful, the collective consumption of food seemed to be a conduit for social engagement and play. Emily and India, for example, shared their experiences of a playful encounter around food which took place during the COVID-19 health pandemic as follows:

“I remember when it was sort of the second wave of lockdown and I was in A&E and, there was a Greggs over the road, they would send sort of breakfast rolls and things in the break room, so we'd all have like a little snack and have like banter” (Emily, p11).

“We had like pizza parties, which were quite good. Everyone definitely enjoyed that, it was good fun. Nurses definitely love their food (laugh), being treated by other people, that's always lovely” (India, p7).

Similarly, Leon described having food on a central table in the A&E Department as follows:

“They have one big table sat in the middle, so like everyone just comes and sits round this one big table so it's a bit like a family dinner, I know you're not all on the same break but there will be 4 or 5 of you at there at some point and everyone is sat there eating and having a laugh” (Leon, p6).

Each account seems to suggest that the gathering of people and the consuming of food, as a communion of sorts, engendered playful practice and promoted social bonding.

Another element of informal play relates to the practice of playful pranks. Many of the participants shared their experiences of pranks which had occurred within the clinical environment. These were most frequently driven by patients or other staff, but in all instances, the participants associated the experience with hedonic feelings.

Emily shared her experience of a prank which occurred on night duty as follows:

“I think on nights it's quite funny, people get up to mischief, cos it's so quiet and all the patients are asleep erm we had this one HCA at (name of Trust) that was telling us something about ghost stories and saying that it was haunted and that kind of thing, you know then you had people jumping out of the linen cupboard with bedsheets all over them, I think that's quite funny too just seeing their reaction” (Emily p13).

Emily went on to say that

“We talked about it for ages after and it was like a couple of months after, I suppose the wards are a bit quieter and there's not as much going on so you can really build your bonds and have a bit more fun. But things like that yeah, it is quite childlike, but it was still quite funny” (giggle) (Emily, p13).

Here, Emily acknowledges that the playful prank mediated positive relationships with peers and recounts the event as being memorable and evocative of childish play.

#### 7.1.2. Structured Informal Play (Games, Quizzes, and Competitions)

Informal play relates to play which was sometimes spontaneous but had an element of structure. Most participants shared an experience of competitive play, and often this was created by other staff. Nancy, for example, shared her experience of a workweek hustle (a synch of a fitbit which allows up to ten people to undertake competitive fitness challenges) as follows:

“They had like a workweek hustle which I joined with my Fitbit, and they were quite competitive, and they would joke and egg you on by saying like “we bet you've tied yours to the dog or you've given yours to your kids to play out with” and stuff like that which was fun” (Nancy, p 2).

Alice shared her experience of playing a game with patients whilst undertaking a physiotherapy placement as follows:

“We used to do erm like group sessions sometimes with some patients. So, at the end of like the group session we did like a game like where you had to throw like a beanbag into a big hole. So, we had like it was like a big board with like all these different targets on it they'd had to throw it in and then we had like 3 patients like going against each other. It was like obviously we're doing things that will really help them, but like they're actually really enjoying themselves at the same time, which is nice to see” (Alice, p9).

Both participants' experiences alluded to the hedonic and eudaimonic benefits of the playful encounter.

The subtheme of informal play captures the essence of play amongst the participants, providing a glimpse into playful practice which has not been observed before in the literature. It is worthy of note that the narrative accounts suggest that the play is purposeful, yielding PWB outcomes.

#### 7.1.3. Subtheme (2): Play with Patients

The topic of patient play had not been identified in the literature review and this subtheme captures the experiences of patient play encountered by the participants. Sometimes driven by the patients themselves, other times coproduced with the participant or the ward/departmental staff, and in all instances, the participants' experience of patient play brought about a number of perceived benefits for both the student and the patient.

Emily and Greta shared their experiences of dressing up, dancing, and having fun with the patients on the ward as follows:

“We were all sort of dressing up and there were Christmas trees and all sorts like that. I think that was really good because it felt like, you know, you were part of the team, everybody was doing their own little thing to like to make the patients day a bit brighter and happier and Christmassy” (Emily, p2).

“One ward I was on, they got like a choir in and they all got up dancing, you could dance with them, so it was nice to see them having some fun as well as me” (Greta, p 2).

Both participants' accounts imply that patient play induced hedonic outcomes, engendering positive relationships and a lifting of mood.

Stuart shared many experiences of patient play in the Radiotherapy Department. In particular, he described a playful encounter where there was a delay on a machine, resulting in the patient's waiting time being extended. Here, he describes having to change the time on the board and the patients changing it back to the original time as follows:

“We had a really long delay so I had to like go up and like change the cards on the board from like 15 minutes to 90 minutes, and this one patient kept coming up and changing it back (laugh) because the waiting time was so long, they were sat there the whole time waiting, so you've got nothing else to do so they were just like playing a game with me the whole time” (Stuart, p12).

He added to this experience by saying that

“It's nice when you've got a patient that is being silly and has like something that they'll talk to you about on a Monday and is a running joke for the rest of the week then. Erm, it makes you feel kind of like oh ok like, they also see me as somebody who's not just here for their treatment” (Stuart, p7).

This playful experience encapsulates the patient creating the play, with Stuart “playing along.” Stuart attributes the playful experience as adding to his self-worth.

Each participant engaged in purposeful play for the benefit of the patients, acknowledging the hedonic and eudaimonic benefits.

### 7.2. Subtheme (3): Social Media Play

This subtheme was created to elucidate the expression of play in the context of the digital platforms used by the participants. During the COVID-19 health pandemic, social media usage such as TikTok grew in popularity [57]. The pandemic witnessed healthcare workers engaged in social media play, as evidenced by the TikTok dances which were prolific at the height of the pandemic. Whilst the pandemic has now abated; it would seem from the participants' experiences that TikTok continues to provide a virtual space within which to play.

Leon describes a TikTok game titled “Rate my Shoes,” which he played with fellow students whilst on clinical placement as follows:

“One of our Uni mates did a TikTok-like rating everyone's shoes that they wear for placement, so she's actually like videoing your shoe and then she'd put like 6/10, bit scuffed, doesn't look waterproof and then she'd like mine and she say like 9/10 erm, loses a point for working in boots, but you know like they're protected from urine and blood and wipe clean and all this and that and then stuff like that which was funny” (Leon, p5).

Likewise, Penny shared her experience of being the “filmer” during the creation of a TikTok dance as follows:

“There would always be one person that couldn't get it, they'd think they'd got it and then as you go and film it and everyone would be like “Come on, we've all got it” and there would be one person who hadn't, so kind of making fun of each other. But it was funny to watch because it's kind of chaos and then it all comes together” (Penny, p8).

Greta described TikTok as something fun to do and a way of connecting (asynchronously) with her friends.

“I like to scroll though social media and message my friends. Go through TikTok, stuff like that, I just like to see what my friends are up to, it's just something fun to do” (Greta, p3).

Conversely, Emily discussed using social media as a way to connect with her friends and crucially, ask for advice or support. Here, she recognises the value of talking to her peers who she believes are having similar experiences in the clinical environment.

“I talk to my friends on like Snapchat or Instagram or whatever, you know just maybe saying ooh I've just had a patient with this and I'm not sure what to do or, you know. It's good to ask somebody who's in the same boat as you as a student (pause) WhatsApp helps erm for instance, if you like say “oh, I'm having a bad day” erm somebody will be there to lift you up” (Emily, p11).

### 7.3. Subtheme (4): Covert Play

Unlike the very visible enactment of play discussed previously, the subtheme of covert play captures the participants' shared experience of play which was ostensibly surreptitious in nature. Penny, for example, shared her experience of working on the hospital wards over the bank holidays as follows:

“Working Bank Holidays, Christmas, Easter or whatever is better because you have more time because it's not kind of routine stuff, you have more time to do more fun stuff like that and kind of mess around” (Penny, p2).

Diane and Emily revealed their playful experiences when they were out of view, or when the ward or department was quiet as follows:

“Sometimes there's like, kind of staff banter, you know behind the scenes if you know what I mean” (Diane, p3).

“Nights are definitely a bit quieter and there's not as much going on so you can really build your bonds and have a bit more fun. But things like that (pranks) yeah, it is quite childlike, but it was still quite funny” (Emily, p13).

Likewise, Stuart shared his experience of play when there was a gap between the patients in the Radiotherapy Department as follows:

“There's little mechanisms of when maybe we don't have a patient for a while as there are little protocols to follow like they have to drink a certain amount of water and we have 5 minutes or so where we do silly little games and stuff or like silly stuff” (Stuart, p23).

The participants acknowledged that the routine of the ward may afford opportunities to engage in play. This type of ludic activity is likely to be intrinsically motivated and may be related to a desire to break boredom, lift mood, or simply have fun. There was certainly a sense from the shared experiences that each participant enjoyed having fun in the workplace and attributed it to a number of hedonic benefits.

## 8. GET (2): The Clinical Playground

As with all playgrounds, there are players, rules, and curators [58, 59] and the clinical playground is no exception. This GET was created to illuminate the reader to the factors which facilitate or limit play and to capture an appreciation of who the players and instigators of play are in the healthcare organisational context. When undertaking the analysis, there was a sense from the participants' lived experiences that some clinical staff played more than others, and that the participants were sensitive to, or aware of a hierarchical and professional dynamic in the clinical environment, and how this influenced the creation of play or (more often) limited playful practice. There was a sense that the participants experienced tension between being a healthcare professional and in engaging in the practice of play. The subthemes have been created to draw the reader's attention to the subtle, rarely articulated factors which relate to the unwritten rules of play within the healthcare context.

### 8.1. Subtheme (1): The Big Personalities

The notion of certain personalities being the creators of the play was expressed frequently by the participants. The subtheme “the big personalities” was produced to highlight the instigators and enablers of play in the clinical playground to the reader. Emily, Penny, and Leon each share their experience of the personalities on the ward or department as follows:

“Like a lot of the junior doctors are on the ward, cos I see them a lot, you'd kind of know who you could have a laugh with but then someone there would be just people that like had big personalities, you go on a ward and there's always that really loud nurse, who makes everyone laugh and is really funny and will kind of get everyone involved in whatever” (Penny, p6).

“I think sometimes staff might have a big personality, that might be a nurse or a physio or an HCA or whatever, I think it depends on the individual people. I think most wards have one” (Emily, p7).

Both accounts recognise that particular individuals drive playful practice. Leon, Greta, and Alice also share their common experience of certain personalities driving play.

“I feel like there's certain people who give off like a certain aura and you know when they walk in and you're like almost laughing already and they've not even said anything” (Leon, p17).

“I can think of one woman who was really funny, but you know when you can't put your finger on what she's done (giggle). I can't think. She's made really good relationships on the ward, she's known as the jokey one” (Greta, p6).

“like the outgoing kind of ones start the banter, they're like quite funny anyways, so they just like to make other people laugh and stuff like that” (Alice, p13).

Their shared experiences acknowledge that certain individuals, particularly those with a certain personality, drive playful practice and there is a hint too, that they expect to meet such people in the clinical playground.

#### 8.1.1. Subtheme (2): The Pecking Order

As discussed previously, students undertaking clinical programmes are invariably exposed to the traditions and rituals of the ward or department where they undertake their clinical learning. Arguably, they are inescapably subjected to inherent hierarchies in the healthcare setting as they develop their professional identity and their position within the hierarchy. This subtheme captures the pecking order in the context of limiters and facilitators of play. Each participant contributed to this subtheme, and the notion of seniority expressed as clinical banding was threaded throughout each of the participant's experiences. Clinical banding (1–9) relates to the UK pay structure in the NHS; the higher bands relate to increased salary and position.

Emily shared her experience of having a playful encounter with senior clinical staff as follows:

“If you're talking to a doctor and having a joke then you feel like they're just human like you are (pause). You know, it gets rid of the ranking system or maybe like the ward manager, they might be band 7 and you might think ooh, they're really important and I need to be careful what I'm saying but if you can have a bit of a joke and a laugh, we can do that together or erm, we can help each other out and I think it makes everyone seem a bit more human” (Emily, p9).

Emily acknowledges the seniority of the doctor and ward manager and alludes to being cautious in their presence, perhaps recognising her position as a student as being inferior to the doctor. She seems to attribute the enactment of play as flattening the hierarchy and contributing to a shared sense of working together. This notion of clinical banding and how it influenced the participants' behaviour was shared by Nancy and Leon, who recognised that opportunities to play were perhaps limited in the presence of the more senior clinical staff.

“The band 8b wasn't there all the time, so when the band 8 came, this is what I mean about the band hierarchy, she was very serious and there was a slightly different tone to the day” (Nancy, p8)

“I've seen band 7's come out of their room, and everyone will sort of like quieten down from what they're doing (playing) and be like head down and do some paperwork” (Leon, p8)

They both acknowledge that the presence of a senior member of staff influenced their behaviour and Leon's account seems to allude to a different type of play, whereby he plays a game of pretence (pretending to be busy) when band 7 is present. Conversely, Stuart's experience acknowledges his junior position in relation to band 8, and again he recognises that his professional behaviour may need to be modified on account of band 8 potentially interviewing him for a job at some point in the future, however, he too seems to allude to play as flattening the hierarchy.

“It's really strange, I think you would think it would be like the highest band, so maybe like a band 8, and you'd be like I can't say anything, like I need to be professional cos they're potentially the person who's looking at me if I apply, so you'd think it would be the highest in the hierarchy, but I actually don't think it is” (Stuart, p10).

There was a sense from each of the participants' experience that they might change their playful behaviours in the company of a senior member of clinical staff.

This subtheme offers an insight into the influencing factors of play in the clinical playground and the participants' lived experiences of play suggest that the pecking order is a limiting factor.

#### 8.1.2. Subtheme (3): The Play Paradox

Each of the participants' experiences alludes to play in the clinical playground and being a professional, on the one hand, they defend the playful actions of others and on the other, they defend their own play. This subtheme was created to highlight the tensions which exist between the professional behaviours, incumbent on healthcare professionals and the enactment of play.

Leon and Emily talk about their feelings regarding the TikTok dances during the height of the COVID-19 pandemic. Both seem quick to defend the actions of their peers.

“I remember there were staff dancing down the corridors and it got loads of backlash. Everyone was like why are you not working and this and that, it's like they're having fun for 10 minutes of their day, just let them. I think, as long as the environment's good, you can always have a laugh and there's no harm in having a laugh either is there?” (Leon, p14).

“I think some people took it in a bad sense of like you're not doing your job, you're too busy doing TikTok dances and all. I just saw it from the other side and thought, that's really good that they can take a breather and have a bit of fun with it and then get back out because obviously it was such a bad time for everybody, it was very difficult, very busy” (Emily, p11).

In contrast, Diane talks about the seriousness of radiation treatment and implies that there is a time and a place to play.

“You can't really be off laughing or joking when you have to make sure that you've got the right patient that you're going to give radiation to, do you know what I mean?” (Diane, p2).

Diane's experience seems to confirm that she understands her professional candour by empathising that plays would only take place at certain times. Similarly, Stuart expressed his experience of having fun with patients yet was keen to emphasise how he would remain professional.

“It's nice when you've got a patient that is being silly and has like something that they'll talk to you about on a Monday and is a running joke for the rest of the week then. Again, it's like if I bumped into them, I hope that we could actually like, I don't know how to say it, in a way like remain professional, but kind of like silly with one another” (Stuart, p8).

There was a sense from the participants' shared experiences that the clinical playground can be difficult to navigate. Their lived experiences of play exposed a range of limiting and facilitative factors, offering new empirical insights into a previously unexplored topic of enquiry.

There were many features of PWB expressed by the participants throughout the analysis and subsequent creation of the GETs. The next GET will discuss these in more detail.

## 9. GET (3): Flourishing

Considered to be more than simply feeling happy, flourishing is a pluridimensional construct combining many aspects of psychological wellbeing [8]. This GET therefore captures elements of PWB that the practice of play seemed to engender among the participants. This finding was significant and supports the notion that play, in the context of the organisational setting, brings about features of PWB. This GET draws the reader's attention to the particular elements of wellbeing which were engendered through the playful practice of undergraduate healthcare students.

During the undertaking of the analysis, it was clear that play fulfilled a number of elements of PWB, irrespective of the type of play. Each participant talked about a playful event as being mood-uplifting, providing relaxation, and/or facilitating a psychological reprieve from the stresses and strains of the clinical environment. The participants' unique experiences provided an allegory of how play facilitated the building of relationships and how play enhanced the patient's experience. Their shared experience of learning was seemingly nurtured and fulfilling in a playful environment. The subthemes reflect elements of play which correspond to PWB indicators.

### 9.1. Subtheme (1): Positive Affect

The subtheme of positive affect (PA) is a key flourishing wellbeing indicator, linked to positive emotion [8]. The subtheme of positive affect was created to highlight the participants' shared experiences of hedonic feelings. Regardless of the type of play, each participant's experience alluded to a lifting or brightening of the mood, in other words, enhanced positive affect. Here, Emily and India share their experiences of having banter on the ward and how it positively impacts their mood.

“It (banter) kind of makes you feel a bit happier and a bit more like excited for the day because you know if you start the day with a bit of a giggle then you're thinking it might be a good day today and you're a bit more enthusiastic maybe” (Emily, p9).

“When you're like starting to like calm down and you've got documentation to do just to like to keep the mood up and stuff, I think people start having a bit of banter” (India, P2).

Along with the lifting of mood, each participant experienced feelings of relaxation or a psychological switch-off as a consequence of the play. There was a sense from the shared experiences that the time engaged in play (often, but not limited to break times), afforded the participants the chance to be less serious and “chill out.” Emily, for example, talks about clearing her mind for a little while before going back “out there.” Here, Emily emotionally distances herself from the pressures of the ward environment and engages in play (TikTok).

“To just almost distract yourself from what's happening on the ward, to clear your mind a little bit before you go back out there (TikTok)” (Emily, p11).

Greta shared her experience of staff talking about the ward events during break time which she did not like, as it “stressed her out.” To avoid this, Greta chose to go on her phone instead.

“Er, go to the staff room, sit there and try and chill for a bit (giggle). Scroll through TikTok, share videos and stuff, it's something fun to do” (Greta, p3).

This notion of psychological detachment is defined in the literature as “the absence of something” [60], in other words, not thinking about the job during nonwork time. Engaging in low-effort activities such as social media and play has been shown to reduce stress [61].

Similarly, Leon talks about play as providing a “release.” Here, he talks about a playful encounter with a patient who presented with a “weird” condition which he knew nothing about, and how funny the interaction became between him and the patient who was equally uninformed. He goes on to say that

“I guess when something like that happens (play) it just puts you in good spirits for the rest of the day, erm yeah, it's a nice little release” (Leon, p3).

Penny shared her experience of working in the acute medical unit (AMU) and how play reduced stress as follows:

“Play definitely relieves stress, it makes wherever you are a little less, erm, you can associate it with fun as well as like tragedy almost. Like I generally don't like AMU, actually no, once I got to know everyone, I liked it but early on it was SO STRESSFUL! There's so much going on, so many patients, like people were being brought in all the time and I'd just find it really stressful, but then with the egg and spoon race, and they did have other things, it made it less like intimidating almost cause then you're like oh, all be really busy but then I'll have a laugh and like the people that will be fun” (Penny, p8).

Penny makes the connection that the practice of play made her experience of placement more inclusive, and that fun was permissible despite the business of the unit.

India describes the energizing effects of play and seems to suggest that play influences her mood which subsequently enhances the mood of others.

“I can't explain it, but yeah, it (play) sort of energizes you, gives you a bit more motivation cos once you start off with a good day, things start rolling onwards if you know what I mean, cos if you are in a good mood, you put others in a good mood almost” (India, p8).

Each of the participants' experiences was suggestive of the notion that positive affect was induced by a playful encounter.

### 9.2. Subtheme (2): Relationships and Connectedness

This subtheme was developed to draw the reader's attention to the participants' practice of play, in relation to promoting positive relationships, building connections, and providing the participants with a sense of clinical-cultural belonging.

Emily shared a particular experience of play which occurred during her clinical placement as follows:

“Sometimes you can be on a ward, and you'll be the only student, so it can feel a little bit isolating, but that was like a really good, sort of, technique to make everybody feel that they were still part of the community. Er, they had like a little kitchen, and they had board games and stuff that you could play on your break which was fun, because you know, you get to know other people as well as the people on the ward” (Emily p3).

Here, she acknowledges the isolation that she experienced as a student nurse and how play enhanced her sense of belonging. Penny too, recognises that being a medical trainee can be difficult and states that

“I'm like a trainee, sometimes it's kind of hard like establish yourself in the team or have people know who you are” (Penny p1).

She goes on to talk about her experience of taking part in a ward egg and spoon race as follows:

“It just, it like brings a bit like, it is just fun and it brings a bit more kind of LIGHTNESS to it, but I think it's good in terms of like, erm, I feel like people remember you when you go back in a few weeks, they'll “oh this is the one who run the race or whatever, so it's good in terms of like team building kind of stuff as well I think, erm cos you've got more things to like associate people with other than work” (Penny, p3).

Here, she confirms that the playful experience promoted positive relationships with the clinical team. Likewise, Stuart shared his experience of belonging as a result of play. Here, he comments on being “part of the crowd” and how the practice of play (frequently expressed as silliness and fun) contributed to this.

“for example, when it's playing with staff, you've usually been comfortable for quite a while. It feels like you're almost like in on the crowd. It's like oh, I've been accepted and it's quite respectful, it's like letting you into their little like, not clique, but like their thing. Erm, so I think like yeah, it feels like you belong” (Stuart, p8)

This subtheme supports the notion that play promotes a sense of connectedness between the student and others.

### 9.3. Subtheme (3): Meaning

There was a sense from the shared experiences of the participants that their engagement in patient play brought about a better experience of care, which in turn was personally fulfilling and/or satisfying. This subtheme was particularly emotive and goes beyond the ludic activities discussed previously, in the sense that the participants' experiences were rooted in a desire to improve the patient experience. Each participant acknowledged the patients' vulnerability owing to them being in the hospital and how the practice of play engendered the PWB indicator of meaning.

Stuart recalls an account when he was undertaking a paediatric radiotherapy placement as follows:

“Just because the child is going through radiotherapy treatment doesn't mean it's the end of the world, I mean a lot of the time, it's like a curative radical treatment, so I usually err on the side of caution, like I'll see how friendly they want to be and how like, erm, I'm not going to throw my one-liners out, but if they are, and if its comfortable, then I will” (Stuart, p5).

Here, Stuart deliberates about if and when to initiate play, his decisions are guided by a desire to improve the patients' experience of care. This feeling was echoed by Diane, in her experience of banter-play with a patient which revealed key eudaimonic wellbeing indicators.

“Because they're coming for radiotherapy, some of them can be coming for like 6 weeks, so erm that's what I like (banter). I like that, getting that rapport with the patient, it makes me feel good you know, like it's all worthwhile” (Diane, p2).

There was a sense from the shared experiences of the participants that their engagement in patient play brought about a better experience of care, which in turn was personally fulfilling and/or satisfying. This subtheme was particularly emotive and goes beyond “playing for playing's sake” in the sense that the participants' experiences were rooted in a desire to improve the patient's experience.

### 9.4. Subtheme (4): Positive Clinical Learning Environment

The subtheme “Positive Clinical Learning Environment” relates to flourishing in the context of the practice setting, be it a hospital ward or a department. Arguably related to the PERMA [8] wellbeing indicators of positive emotion and meaning, this distinct subtheme was created to provide the reader with an insight into the learning dimension of the clinical context. Here, the participants' shared experiences revealed how the practice of play fostered a climate where they were able to ask questions in the pursuit of knowledge, relevant to their clinical practice.

Emily, for example, shared her experience of play in contributing to feeling comfortable and more inclined to ask questions as follows:

“It (play) makes you feel more comfortable, because er, obviously you're meant to learn on placement and you're meant to ask questions, so I think if you can have like a bit of fun with the person who you're with that day, I think it makes you feel more comfortable to ask them questions” (Emily, p9).

Nancy's experience of play in relation to the learning environment was particularly powerful.

“I could relax and be yourself a little bit more, and for me that was big because I felt I learned more, because I felt like I could relax in the learning environment and take more on board. I could ask questions and get stuff wrong; I wasn't worried about looking stupid” (Nancy, p4).

Both participants' acknowledge that play cultivated a comfortable space within which they were able to ask questions. Likewise, Diane and India shared their experience of play and how it gave s them confidence and added value to their role.

“If I have a day when we've had good energy, you know we've had good banter, a good laugh these moments have given me the confidence to do this (the job) I just need to get paid” (Diane, p8).

“It sort of just gives you that bit of confidence, a bit more energy to just to take with you, it makes you feel valued as well” (India, p3).

Each of the participants' experiences of play seemed to capture hedonic feelings related to confidence, which in turn promoted an environment conducive to learning.

## 10. Discussion

The PWB and mental health of the undergraduate student population are of growing concern. Play and PWB are emerging topics of empirical endeavour, with a paucity of studies located in the healthcare organisational context. The study findings, however, confirm that undergraduate students engaged in a diverse range of playful activities in the clinical environment, and these would seem to contribute to enhance PWB. The findings related to informal play, particularly the playful practice of banter, were not insignificant. There are limited empirical studies within the existing literature related to banter in the workplace and seemingly dichotomous opinions about whether banter is harmful or promotes the bonding and socialisation of employees [56, 62]. Importantly, there are no empirical studies to date which have examined playful banter amongst undergraduate students in the healthcare context. This may support the notion that certain types of play often go unnoticed by organisations or are hidden or rebellious [63].

The finding related to food as a conduit for social interaction was not particularly significant but provides a unique insight into the favourable conditions which engender play. Previous studies have explored the role of food and eating in the promotion of human connections and play [64, 65]. A study by Dunbar [65], for example, found that social dining, as well as creating and strengthening relationships, yielded significant health benefits. In contrast, but in keeping with the theme of food, the seminal “banana time” study by Roy [66] demonstrated how food as a prop served as a conduit for the initiation of humour and good-natured banter amongst employees.

The characterisation of patient play was a surprising finding from the study. Patient play was not always driven by the participants and was often coproduced with the patient, suggesting perhaps a “play driver” which has previously gone unnoticed in the literature. Arguably, this is due to the fact that play in the healthcare context is an emerging area of empirical inquiry and thus primary data sources are limited. That withstanding, there are a handful of studies, mainly from oncology literature, which have examined patient-initiated humour play [67], however, studies are limited to testing the fidelity of humour as a therapeutic approach to care. Thus, the findings from this study extend to a more sophisticated characterisation of play; the enactment of patient play. Manifested as purposeful, sometimes mischievous, and highly interactive, this aspect of play offers direction for future empirical endeavour.

The notion of play through social media was an experience shared by many of the participants. Sometimes this was asynchronous, other times synchronous, and perhaps reflects a generational mode of playing and socialising. This has not been observed previously and extends the discussion to possible tools through which to achieve psychological wellbeing benefits. There is certainly emerging evidence to suggest that millennials and iGENs engage with social media platforms to play and virtually connect, often to escape offline from psychological troubles [68]. However, there is a difference of opinion in the literature about the benefits of social media usage and how it may adversely affect mental health and wellbeing [69]. That withstanding, it is worthy of the note that the majority of the students in the study would be considered to be iGENs [70], and therefore had the population been from another age range, this type of play may not have been found. Nonetheless, the expression of social media play adds to a sparse body of knowledge around play in the healthcare organisational context.

Likewise, the expression of covert play was a key finding, confirming that the students played in the clinical environment, particularly when the ward or department was less demanding, thus adding another facet to the typology of play. This notion of “play for the sake of play” has been observed in the literature [71], but studies are mostly bound by child-play studies, with no extant empirical studies which have examined play amongst adults in the healthcare organisational context. This finding therefore sheds new light on a misunderstood or perhaps dismissed aspect of play.

The study found a number of facilitating and limiting factors of play, offering some unique empirical insights. Previous organisational play research has mostly focused on the outcomes of play, with little consideration given to the factors (both individual and organisational) which facilitate or limit playful practice [27]. The finding that “big personalities” were the main facilitators of play in the healthcare context had not been explored before and therefore cannot be supported by existing empirical studies. That withstanding, the participants recognised that certain personalities were more likely to engage in or drive playful practice and this would seem to align with the notion of personality traits as a key theoretical perspective. Personality traits have been observed in the literature and are characterised as those individuals who are spontaneous, gregarious, and joyful [72]. There remains a dearth of literature related to drivers of play, with the exception of “manufactured play” located within the realms of serious play and employer-driven play. The findings therefore contribute to a sparse body of knowledge.

Finally, the notion of PWB in the context of play within the healthcare environment has not been empirically examined previously and this study brings new insights, by revealing that the enactment of play would seem to bring about enhanced psychological wellbeing (PWB) in a range of established (PERMA) wellbeing indicators [8]. A key finding of the study was that a positive effect was induced by the practice of play. Captured as a mood-lifting, emotional relief, energizing, or soothing, this finding is aligned to Seligman's [8] PWB indicator of positive emotion. Indeed, positive emotion has been delineated to characterise the features of positive affect into hedonic and eudaimonic wellbeing: hedonic relating to enjoyment and feeling happy, and eudaimonic relating to purposeful engagement [73]. The influence of positive affect on wellbeing and health has been much discussed in the literature over recent decades [74]. It is worthy of note that positive affect (mood) is associated with long-term health and wellbeing [75]. Therefore, the finding that the students' experienced enhanced mood during play was a significant finding.

The study also found that the enactment of play, irrespective of type, would seem to provide an opportunity to detach from the pressures of work and facilitate a psychological reprieve or uplift of mood. This adds new insights into the PWB indicator of positive emotion and is supported by the key theoretical perspectives of psychological detachment. Psychological detachment is defined in the literature as “the absence of something” [60], in other words, not thinking about the job during the nonwork time. Engaging in low-effort activities such as play has been shown to facilitate psychological detachment and reduce stress [61]. Arguably, the findings also accord with the cathartic theoretical perspective of play [76] and relate to playful practice which releases tension and stress, providing psychological relief.

In addition, positive relationships are widely accepted pillars of PWB and are a feature of Seligman's [8] PERMA wellbeing model. It is accepted within extant literature that social connectedness promotes both physical and mental health [77, 78], and indeed the World Health Organization [79] recognises positive relationships as an important social determinant of health. The findings from this study confirm that the enactment of play promoted positive relationships and facilitated interpersonal connections. This finding is supported by studies from the wider literature [80–83] which found that the enactment of play, albeit predominantly employer-driven, facilitated person-to-person bonds and team spirit and human connections. The unique insights related specifically to the healthcare organisational setting amongst undergraduate students have not been observed before.

The PWB indicator of meaning was not found in the literature and is possibly due to the organisational context where the studies were undertaken. As mentioned previously, the healthcare organisational environment is distinct from other business sectors, and arguably healthcare personnel seek caring roles which offer a sense of personal worth. This study found that the practice of play afforded the students a sense of personal satisfaction, which in turn optimised the patient experience, engendering purpose and self-worth.

Finally, the study found that the enactment of play created an environment where the students were more comfortable and therefore more receptive to learning. This was an interesting finding, and the topic of play and learning in the clinical environment has been observed in the literature in terms of the sociocultural theory of human learning [84]. Sociocultural theory and situated learning emphasise how social and culturally organised activities influence cognition and learning [85]. Indeed, Kolb and Kolb [86] 47 states that “Play exemplifies one of the highest forms of experiential learning.” However, it is rarely used with adults, and this perhaps gives rise to the notion that play amongst adults is yet to be fully explored as both an agent for enhanced learning as well as the promotion of PWB. This finding may also be redolent of the “hidden curriculum,” whereby the students learn the rules of play as they go along. The hidden curriculum was first observed in the literature in the 1980s [87] and defined more recently by Raso et al. [88] 989 as a “learning dimension made up of culturally acquired, unintended lessons.” However, this was beyond the scope of the study and offers a new direction for future empirical inquiry.

## 11. Implications and Recommendations for Practice

It is perhaps time to redefine play in the healthcare and educational organisational context and consider playful practice as an extension of enhanced communication skills, thereby legitimising play as a skill befitting of a healthcare professional. Furthermore, the reappraisal of the spectrum of understanding about what being a healthcare professional entails will pave the way for a new thinking. By creating a shared understanding of what is meant by play, clinicians and educators can offer students and healthcare workers permission to play, outlining the context within which it is permissible. As discussed above, by redefining play as an enhanced communication skill, there is the potential for it to be taught within the higher education institution (HEI) setting. It is recommended that the vehicle to do this would be through simulated teaching. Simulation has gained popularity over the recent years and is now a commonplace within medical, nursing, and AHP curricula [89]. However, the concept of simulation, which is to create a safe and real-life learning environment for students [90], has tended to focus on the acquisition of technical clinical skills, evidenced by the increased use of augmented and virtual reality devices [91]. Thus, by introducing the “softer clinical skills” to the simulation curriculum, there is an opportunity for play to be incorporated into advanced communication skills training.

There is a critical mass of students currently in higher education, most of whom would be considered to be “iGENs.” Uniquely different, this cohort of students is more playful and has never known life without a smartphone [92]. This is significant, and as discussed in the findings, the students used their digital devices to play, socialise, and connect, albeit asynchronously at times. It is therefore recommended that both the healthcare and education providers should consider how smartphones and digital devices can be used to promote the wellbeing of students.

The findings from this research indicate that students often played during off-clinical time, such as breaks. Therefore, it is recommended that dedicated spaces be created for students and healthcare workers to eat, socialise, and play. Furthermore, it is recommended that such spaces are resourced with play props such as board games, thereby facilitating detachment from the demands of the clinical environment. This recommendation can also be extended to education providers in terms of providing and resourcing spaces for students to play and emotionally detach from the pressures of university life. Moreover, the characterisations of play and the factors which facilitate and/or limit play in the healthcare setting are incomplete and this warrants further empirical investigation. Finally, the findings from this study offer a new direction for play as a facilitator of PWB for undergraduate healthcare students.

Play and PWB are emerging topics of empirical endeavour, and this study has confirmed that an empirical relationship exists between the two in a distinct population. The findings that play enhanced the PERMA [8] wellbeing indicators of positive emotion, relationships, and meaning lend weight to future empirical inquiry into other elements of wellbeing such as engagement and accomplishment. Furthermore, the notion of the patient as a “play driver” has been overlooked in the literature and this is worthy of future empirical exploration. Moreover, the characterisations of play and the factors which facilitate and/or limit play in the healthcare setting are incomplete and this too warrants empirical investigation.

## 12. Limitations

First, the study was limited by the fact that the participants' lived experiences of play occurred during the time of a global health pandemic; however, the insights gleaned shed light on previously unexplored topics of inquiry. Secondly, it could be argued that the sample size was a limitation of the study, however, since the study was qualitative in design, a sample of nine was methodologically congruent with IPA studies and arguably produced a depth and richness of data which could not have been amassed by using a larger sample size. Thirdly, the data were collected via one-to-one semistructured interviews. It is recognised within the extant literature that there may be an imbalance of power between the interviewer and the interviewee [93, 94]. In other words, the interviewer knows what they are going to ask, and the interviewee does not. Careful preparation and adherence to the ethical principles of prevention of harm mitigated this.

## 13. Conclusion

The study confirmed that undergraduate medicine and healthcare students engaged in a diverse range of playful activities in the clinical environment, resulting in the creation of a typology of play, capturing the expression of play amongst a previously untested population, and therefore adding an original contribution to the existing play literature. By providing unique insights into the factors which facilitate and limit the enactment of play in the healthcare organisational context, the study findings add to a dearth of existing literature and provide some gleanings into future empirical endeavour. Crucially, the study found that the enactment of play induced key hedonic and eudaimonic PWB benefits, ranging from positive affect to improved relationships, a sense of meaning, and a positive learning environment, offering original empirical insights. These findings have not been observed previously and shine a conceptual light on a previously unknown phenomenon.

## Figures and Tables

**Table 1:** Sample.

Participant	Age	Undergraduate Programme
Greta	20	Nursing
India	21	Nursing
Stuart	22	Therapeutic radiography
Nancy	42	Physiotherapy
Diane	31	Therapeutic radiography
Emily	21	Nursing
Leon	20	Nursing
Alice	20	Physiotherapy
Penny	25	Medicine

**Table 2:** Interview topic guide.

Technical set up/preparation		

Check recording equipment		
Pen/paper		

Setting the background		

Introduction		
Informed consent		
Check participants' programme of study and year		
Confidentiality, safeguarding, and professional concerns		
Reiterating the scope of the study		

Icebreaker		

What do you enjoy about your clinical placement?		

Topic guide		

Questions		Prompts and probes

1	Can you tell me about a time when something fun/playful happened at work?	How did that come about? (What was happening/happened on the ward/placement at that time?)
		When did this happen?
		What else is there about the situation which made it playful?
		What happened as a result?
		What happened afterwards?
		How did it impact you?
		Who else did it impact?

2	How did it affect you?	How did you feel?
		How long did it last?

3	What was your next example?	How does this differ from the previous example?

4	Tell me about other playful examples which are different?	Remember to let the silence in

Winding down		

Thank you		
Any questions?		
Emotional check-in ^*∗*^Advise that a follow-up call will happen in the next week		

**Table 3:** Master table of GETs.

PETs	Direction of analytical movement	GET (1)	Subthemes

Teasing, jokes, banter, competitions, board games, pranks, and food	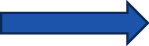	Playtime (expression of play)	Subtheme 1
			Informal play
			(a) Unstructured
			(b) Structured
Having fun with patients	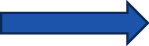		Subtheme 2
			Patient play
TikTok and Snapchat to play and connect	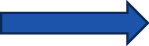		Subtheme 3
			Social media play
Behind the scenes, when the ward/department is quiet	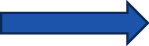		Subtheme 3
			Covert play

PETs	Direction of analytical movement	GET (2)	Subthemes

Individual staff and their personal characteristics instigating play	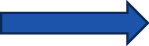	The clinical playground (facilitative and limiting factors)	Subtheme 1
			“Big personalities”
Need to be careful around the band 6's and 7s	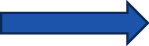		Subtheme 2
			“The pecking order”
There is a time and a place to play	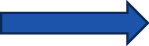		Subtheme 3
			“The play paradox”

PETs	Direction of analytical movement	GET (3)	Subthemes

Play lightning/boosting mood	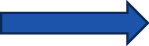	“Flourishing” (psychological wellbeing)	Subtheme 1
Energizing/soothing effects of play			Positive affect
In with the crowd “acceptance”	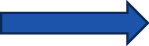		Subtheme 2
Team building			Relationships and connectedness
Play makes the patients' episode of care more comfortable, leading to a sense of purpose and job satisfaction	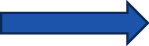		Subtheme 3
			Meaning
Play opens up a space to ask questions	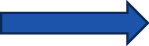		Subtheme 4
			Positive clinical learning environment

## Data Availability

The narrative data used to support the findings of this study are included within the article.

## References

[1] De Simone S. (2014). Conceptualizing wellbeing in the workplace. *International Journal Of Business And Social Science*.

[2] Buffet M. A., Gervais R. L., Liddle M., Eekelaert L. (2013). *Wellbeing At Work: Creating a Positive Work Environment. Literature Review. European Agency for Health at Work*.

[3] Bevan S. (2010). *The Business Case for Employees Health and Wellbeing*.

[4] Henry J., Haworth J., Hart G. (2007). Positive psychology and the development of wellbeing. *Wellbeing*.

[5] Kun Á., Balogh P., Gerákné Kranz K. (2017). Development of the work-related well-being questionnaire based on Seligman’s PERMA model. *Periodica Polytechnica, Social and Management Sciences*.

[6] Winefield H., Gill T., Taylor A., Pilkington R. (2012). Psychological well-being and psychological distress: is it necessary to measure both?. *Psychology of Well-Being: Theory, Research and Practice*.

[7] Deci E. L., Ryan R. M. (2008). Hedonia, eudaimonia, and well-being: an introduction. *Journal of Happiness Studies*.

[8] Seligman M. E. P. (2011). *Flourish, a New Understanding of Happiness and Wellbeing – and How to Achieve Them*.

[9] Huppert F. A. (2009). Psychological well-being: evidence regarding its causes and consequences. *Applied Psychology: Health and Well-Being*.

[10] Kinman G., Teoh K., Harriss A. (2020). Supporting the well-being of healthcare workers during and after COVID-19. *Occupational Medicine*.

[11] Søvold L. E., Naslund J. A., Kousoulis A. A. (2021). Prioritizing the mental health and well-being of health workers: an urgent global public health priority. *Frontiers in Public Health*.

[12] Janse van Vuuren E. C., Bodenstein K., Nell M. (2018). Stressors and coping strategies among physiotherapy students: towards an integrated support structure. *Health SA, Journal of Interdisciplinary Health Sciences*.

[13] Pryjmachuk S., Richards D. (2007). Predicting stress in pre-registration nursing students. *British Journal of Health Psychology*.

[14] Cannon T. (2017). Promoting Student wellbeing. https://www.redbrickresearch.com/2017/11/30/promoting-student-wellbeing/.

[15] Office for National Statistics (2020). *Coronavirus and the Impact on Students in Higher Education in England: September to December 2020*.

[16] Maben J., Bridges J. (2020). Covid-19 supporting nurses psychological and mental health. *Journal of Clinical Nursing*.

[17] Abram M. D., Jacobowitz W. (2021). Resilience and burnout in healthcare students and inpatient psychiatric nurses; a between-groups study pf two populations. *Archives of Psychiatric Nursing*.

[18] Cairns P., Aitken G., Pope L. (2021). Interventions for the well-being of healthcare workers during a pandemic or other crisis: scoping review. *BMJ Open*.

[19] Verenikina I. M., Hasan H. M. (2010). The importance of play in organisation. *Faculty of Health and Behavioural Sciences Papers (Archive)*.

[20] Dodgson M. (2016). Innovation and play. *Innovation*.

[21] Petelczyc C. A., Capezio A., Wang L., Restubog S. L. D., Aquino K. (2018). Play at work: an integrative review and agenda for future research. *Journal of Management*.

[22] Van Vleet M., Feeney B. C. (2015). Young at heart: a perspective for advancing research on play in adulthood. *Perspectives on Psychological Science*.

[23] Sørensen B., Spoelstra S. (2011). Play at work: continuation, intervention and usurpation. *Organization*.

[24] Everett A. (2005). Benefits and challenges of fun in the workplace. *Library Leadership and Management*.

[25] Karl K., Peluchette J. V., Harland L. (2005). Attitudes toward workplace fun: a three sector comparison. *Journal of Leadership and Organizational Studies*.

[26] Berg D. H. (2001). The power of a playful spirit at work. *Journal for Quality and Participation*.

[27] Celestine N. A., Yeo G. (2021). Having some fun with it: a theoretical review and typology of activity‐based play‐at‐work. *Journal of Organizational Behavior*.

[28] Tökkäri V. (2014). Organizational play: within and beyond managing. *Qualitative Research in Organizations and Management: An International Journal*.

[29] Spraggon M., Bodolica V. (2018). A practice-based framework for understanding (informal) play as practice phenomena in organizations. *Journal of Management and Organization*.

[30] West S. E., Hoff E., Carlsson I. (2016). Play and productivity. Enhancing the creative climate at workplace meetings with play cues. *American Journal of Play*.

[31] Chernomas W. M., Shapiro C. (2013). Stress, depression and anxiety among undergraduate nursing students. *International Journal of Nursing Education Scholarship*.

[32] Mitchell A. (2020). The perceived psychological stressors and coping behaviours in university students, on a pre-registration programme. *The Journal of Mental Health Training, Education and Practice*.

[33] General Medical Council (2015). *Supporting Medical Students with Mental Health Conditions*.

[34] Brooke T., Brown M., Orr R., Gough S. (2020). Stress and burnout: exploring postgraduate physiotherapy students’ experiences and coping strategies. *BMC Medical Education*.

[35] Eslick G., Raj V. (2002). Occupational stress amongst radiographers: does working in private or public practice make a difference?. *Radiography*.

[36] Ibikunle P., Amah E., Useh U. (2016). Prevalence and pattern of burnout syndrome among healthcare professionals in a university teaching hospital. *Tropical Journal of Medical Research*.

[37] Labrague L. J., McEnroe-Petitte D. M., Gloe D., Thomas L., Papathanasiou I. V., Tsaras K. (2017). A literature review on stress and coping strategies in nursing students. *Journal of Mental Health*.

[38] Rafati F., Nouhi E., Sabzevari S., Dehghan-Nayeri N. (2017). Coping strategies of nursing students for dealing with stress in clinical setting: a qualitative study. *Electronic Physician*.

[39] Bhattacharyya P., Jena K. L., Pradhan S. (2019). Resilience as a mediator between workplace humour and well-being at work: an enquiry on the healthcare professionals. *Journal of Health Management*.

[40] Christopher J. (2015). An introduction to black humour as a coping mechanism for student paramedics. *Journal of Paramedic Practice*.

[41] Danzinger R. (2018). He who laughs last laughs best. Humor as a consolation for the dying and their relatives. http://www.rainerdanzinger.at.

[42] Watson K. (2011). Gallows humor in medicine. *Hastings Center Report*.

[43] Nunstedt H., Eriksson M., Obeid A., Hillström L., Truong A., Pennbrant S. (2020). Salutary factors and hospital work environments: a qualitative descriptive study of nurses in Sweden. *Biomed Central Nursing*.

[44] Healy C. M., McKay M. F. (2000). Nursing Stress: the effects of coping strategies and job satisfaction in a sample of Australian Nurses. *Journal of Advanced Nursing*.

[45] Collicutt J., Gray A. (2012). A Merry Heart Doeth good like Medicine: humour, religion and wellbeing. *Mental Health, Religion and Culture*.

[46] Ghaffari F., Dehghan-Nayeri N., Shali M. (2015). Nurses’ experiences of humour in clinical settings. *Medical Journal of the Islamic Republic of Iran*.

[47] Smith J. A., Flowers P., Larkin M. (2022). *Interpretative Phenomenological Analysis*.

[48] Smith J. A., Nizza I. (2022). *Essentials of Interpretative Phenomenological Analysis*.

[49] Charlick S., Pincombe J., McKellar L., Fickler A. (2016). Making sense of participants experiences: interpretive phenomenological analysis in midwifery research. *International Journal of Doctoral Studies*.

[50] Larkin M., Thompson A. R., Harper D., Thompson A. R. (2011). Interpretive phenomenological analysis in mental health and psychotherapy research. *Qualitative Research Methods in Mental Health and Psychotherapy: A Guide for Students and Practitioners*.

[51] Roberts K., Dowell A., Nie J.-B. (2019). Attempting rigour and replicability in thematic analysis of qualitative research data: a case study of code book development. *BMC Research Methodology*.

[52] Yardley L. (2000). Dilemmas in qualitative health research. *Psychology and Health*.

[53] Berend B., Voght D., Brohm-Badry M. (2020). Positive emotions and flourishing are resilience factors for stress symptoms. *International Journal of Stress Promotion and Wellbeing*.

[54] Oxford University Press (2022). Oxford english dictionary. https://www.oed.com.

[55] Hay J. (1994). Jocular abuse patterns in mixed-group interaction. *Wellington Working Papers in Linguistics*.

[56] Plester B. A., Sayers J. (2007). Taking the piss: functions of banter in the IT industry. *International Journal of Humor Research*.

[57] Chapple C. (2020). TikTok crosses 2 billion downloads after best quarter for any app ever. https://sensortower.com/blog/tiktok-downloads-2-billion.

[58] Salen K., Zimmerman E. (2004). *Rules of Play: Game Design Fundamentals*.

[59] Huizinga J. (1949/2016). Homo ludens. A study of the play-element in our culture. *Kettering: Anglicopress.com*.

[60] Sonnentag S., Fritz C. (2015). Recovery from job stress: the stressor detachment model as an integrative framework. *Journal of Organizational Behaviour*.

[61] Ragsdale J. M., Beehr T. A., Grebner S., Han K. (2011). An integrated model of weekday stress and weekend recovery of students. *International Journal of Stress Management*.

[62] Dynel M. (2008). No aggression, only teasing: the pragmatics of teasing and banter. *Lodz Papers in Pragmatics*.

[63] Henricks T. S. (2014). Play as self-realisation: toward a general theory of play. *American Journal of Play*.

[64] Batat W., Peter P. C., Moscato E. M. (2019). The experiential pleasure of food: a savouring journey to food well-being. *Journal of Business Research*.

[65] Dunbar R. I. M. (2017). Breaking bread: the function of social eating. *Adaptive Human Behaviour and Physiology*.

[66] Roy D. F. (1959). Banana time: job satisfaction and informal interaction. *Human Organization*.

[67] Adamle K. N., Ludwick R., Zeller R., Winchell J. (2008). Oncology nurses response to patient-initiated humour. *Cancer Nursing*.

[68] Yadav P. G., Rai J. (2017). The generation Z and their social media usage: a review and a research outline. *Global Journal of Enterprise Information System*.

[69] Chotpitayasunondh V., Douglas K. (2018). The effects of ‘Phubbing’ on social interaction. *Journal of Applied Social Psychology*.

[70] Wiedmer T. (2015). Generations do differ: best practices in leading traditionalists, boomers and generations X, Y and Z. *The Delta Kappa Gamma Bulletin*.

[71] Besio S., Bulgarelli D., Syancheva-Popkostadinova V. (2022). *Play Development in Children with Disabilities*.

[72] Barnett L. A. (2007). The nature of playfulness in young adults. *Personality and Individual Differences*.

[73] Fredrickson B. L. (2001). The role of positive emotions in positive psychology: the broaden-and-build theory of positive emotions. *American Psychologist*.

[74] Brondino M., Raccanello D., Burro R., Pasini M. (2020). Positive affect over time and emotion regulation strategies: exploring trajectories with latent growth mixture model analysis. *Frontiers in Psychology*.

[75] Steptoe A., O’Donnell K., Marmot M., Wardle J. (2008). Positive affect, psychological well-being, and good sleep. *Journal Of Psychosomatic Research*.

[76] Ellis M. J. (1973). *Why People Play*.

[77] Duke L. H. (2017). The importance of social ties in mental health. *Mental Health and Social Inclusion*.

[78] Umberson D., Karas Montez J. (2010). Social relationships and health: a flashpoint for health policy. *Journal of Health and Social Behavior*.

[79] World Health Organisation (2013). *Review of Social Determinants and the Health Divide in the WHO European Region: Final Report*.

[80] Sakr C., Zotti R., Khaddage-Soboh N. (2019). The impact of implementing fun activities on employee’s engagement. *International Journal of Organizational Analysis*.

[81] Han H., Kim W., Jeong C. (2016). Workplace fun for better team performance: focus on frontline hotel employees. *International Journal of Contemporary Hospitality Management*.

[82] Georganta K., Montgomery A. (2016). Exploring fun as a job resource: the enhancing and protecting role of a key modern workplace factor. *International Journal Of Applied Positive Psychology*.

[83] Wijewardena N., Samaratunge R., Härtel C., Kirk-Brown A. (2016). Why did the emu cross the road? Exploring employees’ perception and expectations of humor in the Australian workplace. *Australian Journal of Management*.

[84] Vygotsky L. S. (1978). *Mind in Society: The Development of Higher Psychological Processes*.

[85] Black R. W., Reich S. M. (2011). Affordances and constraints of scaffolded learning in a virtual world for young children. *International Journal of Game-Based Learning*.

[86] Kolb A. Y., Kolb D. A. (2010). Learning to Play, Playing to Learn: case study of ludic learning space. *Journal of Organizational Change Management*.

[87] Bell J. (1984). The hidden curriculum. *Nursing Mirror*.

[88] Raso A., Marchetti A., D’Angelo D. (2019). The hidden curriculum in nursing education: a scoping study. *Medical Education*.

[89] Motola I., Devine L. A., Chung H. S., Sullivan J. E., Issenberg S. B. (2013). Simulation in healthcare education: a best evidence practical guide. AMEE Guide No. 82. *Medical Teacher*.

[90] Cook D. A., Hatala R., Brydges R. (2011). Technology-enhanced simulation for health professions education: a systematic review and meta-analysis. *Journal of the American Medical Association*.

[91] Gunn T., Jones L., Bridge P., Rowntree P., Nissen L. (2018). The use of virtual reality simulation to improve technical skill in the undergraduate medical imagining student. *Interactive Learning Environments*.

[92] Ozkan M., Solmaz B. (2015). Mobile addiction of generation Z and its effects on their social lifes: (an application among university students in the 18-23 age group). *Social and Behavioral Sciences*.

[93] Råheim M., Magnussen L. H., Sekse R. J. T., Lunde Å., Jacobsen T., Blystad A. (2016). Researcher–researched relationship in qualitative research: shifts in positions and researcher vulnerability. *International Journal of Qualitative Studies on Health and Well-Being*.

[94] Brinkmann S., Kvale S. (2006). Confronting the ethics of qualitative research. *Journal of Constructivist Psychology*.

